# Sources of Variability in Intracranial Aneurysm Model Reconstruction and Evaluation: A Systematic Investigation

**DOI:** 10.1007/s13239-026-00841-1

**Published:** 2026-05-07

**Authors:** Jared T. Chong, Hang Yi, Alex E. Wang, Cindy Ju, Luke Bramlage, Bryan Ludwig, Zifeng Yang

**Affiliations:** 1 Department of Mechanical and Materials Engineering, Wright State University, Dayton, OH 45435 USA; 2 Centerville High School, 500 E Franklin St, Centerville, OH 45459 USA; 3 Upper Arlington High School, 1625 Zollinger Rd, Upper Arlington, OH 43221 USA; 4 Division of NeuroInterventional Surgery, Department of Neurology, Wright State University/Premier Health—Clinical Neuroscience Institute, 30E. Apple St., Dayton, OH 45409 USA; 5 Boonshoft School of Medicine, Wright State University, Dayton, OH 45435 USA

**Keywords:** Aneurysm, Model, Reconstruction, Threshold

## Abstract

**Purpose:**

The intracranial aneurysm (IA) model reconstruction is critical for pathophysiology diagnosis and computational simulations. This study aimed to quantify the impact of segmentation thresholds and software platforms on the reconstruction of IA geometry as well as the impact of inter-user variability on the assessment of morphology.

**Methods:**

600 IA models were reconstructed from 100 patient DSA datasets using Materialise Mimics and 3D Slicer at three grey value (GV) thresholds; 1000, 1500, and 2500. Geometric measurements were performed in 3-matic by three users. Measurements included vessel diameters and aneurysm morphology parameters. Mimics, the 2500 GV threshold, and the most experienced user (R1) served as baselines for comparison. Normality was evaluated using Shapiro-Wilk tests, and statistical differences were assessed with paired t-tests and relative percent differences.

**Results:**

All anatomical regions showed statistically significant geometric variation across software and threshold. Model evaluation showed potential statistically significant variation between users. Models from 3D Slicer were consistently smaller than those from Mimics with percent differences ranging from − 1.27 to − 4.38% (all p < .05). Lower thresholds produced consistently larger models; decreasing from 2500 to 1000 GV increased average diameters by up to 15.9%, depending on specific region. User-related variability was most pronounced in the least experienced user, with size measurements deviating by up to 22.67% from the baseline.

**Conclusion:**

Segmentation software, threshold selection, and user interaction each introduce meaningful and statistically significant variability into IA model geometry and evaluation. Standardization of segmentation protocols—especially threshold values and operator training—is essential to improve reproducibility.

**Supplementary Information:**

The online version contains supplementary material available at 10.1007/s13239-026-00841-1.

## Introduction

Accurate reconstruction and evaluation of intracranial aneurysms (IA) models is essential for medical diagnosis, treatment planning, and research applications such as morphology biomarkers assessment and hemodynamic analysis using computational fluid dynamics (CFD) simulations. Previous studies have evaluated the accuracy of intracranial aneurysm segmentation and model reconstruction using a variety of imaging modalities, segmentation techniques, and validation approaches [1–8]. While validation studies are common, few isolate the effects of specific parameters such as threshold selection, software platform, and operator variability on reconstructed geometry.

The proposed study seeks to fill the gaps in previous works by offering systematically researched, statistically significant results that quantify how sources of uncertainty contribute to differences in model geometry, and, in doing so, improve the standardization of IA model reconstruction based on rotational digital subtraction angiography (DSA) a fluoroscopic technique that enhances visualization of vascular structures by subtracting pre-contrast images from contrast-enhanced images. By studying the geometry of a sufficiently large sample size of reconstructed IA models (600 models from 100 patients) while also introducing and documenting potential sources of discrepancies (i.e., different software, different thresholds, different users), a statistically significant level of variability was shown to exist within IA model reconstruction and evaluation.

## Methods

For this study, a total of 600 IA models were reconstructed using two widely utilized medical image processing platforms: Mimics 25.0 (Materialise NV, Leuven, Belgium) and 3D Slicer 5.6.2 (Brigham and Women’s Hospital, Massachusetts, USA). DSA scans from 100 de-identified patients diagnosed with IAs were obtained in the form of DICOM files from Miami Valley Hospital (Premier Health), in Dayton, OH, USA. All cases were selected based on the availability of high-quality DSA imaging for cerebral aneurysms suitable for 3D reconstruction. For each case, 390 2D sliced images were used to reconstruct the aneurysmal model. The datasets had an isotropic spatial resolution of approximately 0.29 × 0.29 × 0.29 mm^3^. Acquisition settings were consistent across all cases. The IA models for each patient case were reconstructed using distinct lower threshold values of 1000, 1500, and 2500 GV; thus, three models were constructed under each image processing platform accordingly for a total of six models per case. It is important to clarify that GV refers to the voxel intensity values displayed by each software platform following application of DICOM rescale parameters. While these values are derived from Hounsfield Units (HU), they represent software-specific intensity domains.

In typical clinical workflows, segmentation thresholds are often adjusted interactively while visually evaluating vessel contour. In contrast, fixed threshold values were used in the present study to systematically evaluate how threshold selection influences aneurysm morphology independent of operator driven contour adjustments. The lower threshold values of 1000, 1500, and 2500 GV were selected based on empirical comparison of reconstructed vascular geometries with patient-specific 3D DSA images by two independent radiologists/physicians. Thresholds below 1500 GV tended to introduce excessive noise while higher thresholds resulted in loss of vascular detail including smaller vessels. This range represented a balance between noise reduction and preservation of anatomical detail.

**Fig 1:**
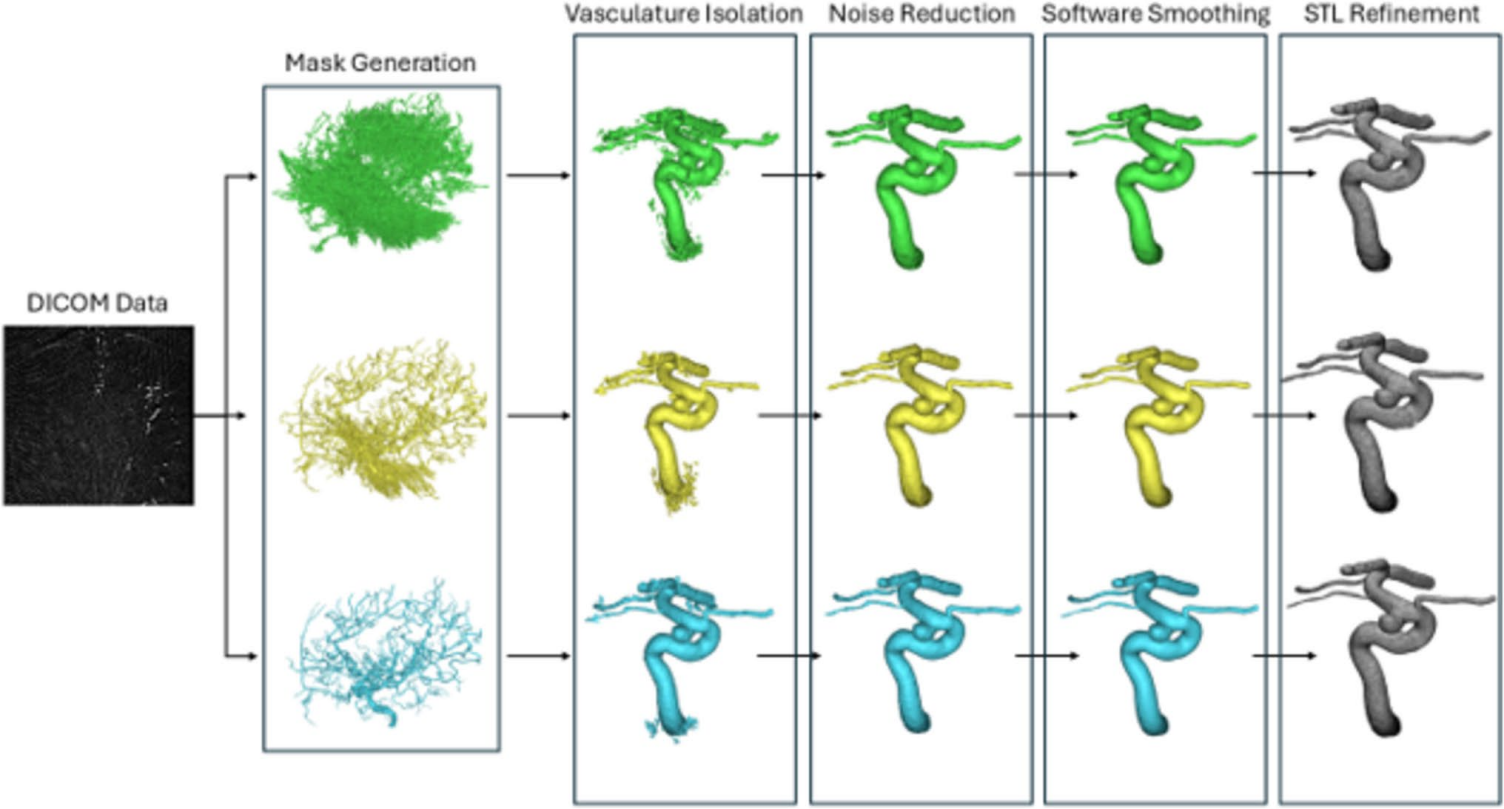
A representative patient-specific aneurysm case reconstructed using the standardized workflow for IA model reconstruction

The standardized reconstruction workflow as shown in Fig. 1, consisted of the following steps: importing DICOM data, thresholding masks, isolating cerebral vasculature, reducing image noise, applying software-unique smoothing algorithms, and post-processing STL refinement. Final models were then evaluated for geometric consistency.

Geometric measurements were conducted independently by three well-trained individuals (i.e., R1, R2 and R3) to assess geometric differences between the two software as well as inter-user variability. R1 is a graduate student with approximately one year of experience with both software packages; R2 and R3 are individuals with approximately six months and one month of experience, respectively. The resulting data sets were statistically analyzed to determine the extent and significance of variation introduced by threshold selection, software platforms, and user expertise.

### Model Reconstruction

Model reconstruction followed the benchmark procedures detailed by Yi et al. in previous publications [9]. Model reconstruction began by importing DICOM files into the designated medical image processing software environment. For each case, the lower threshold value for segmentation was manually set to 1000, 1500, or 2500 GV depending on the model variant; these thresholds fall within a commonly used range for model reconstruction. The upper threshold value was left at the default, automatically determined setting unique to each software platform. Prior to the application of any noise reduction techniques, major cerebral arteries were first identified and segmented to ensure that important vascular structures were not inadvertently removed during the noise reduction process. This step was particularly critical for cases with substantial background noise where vessel preservation required careful anatomical scrutiny.

**Fig 2:**
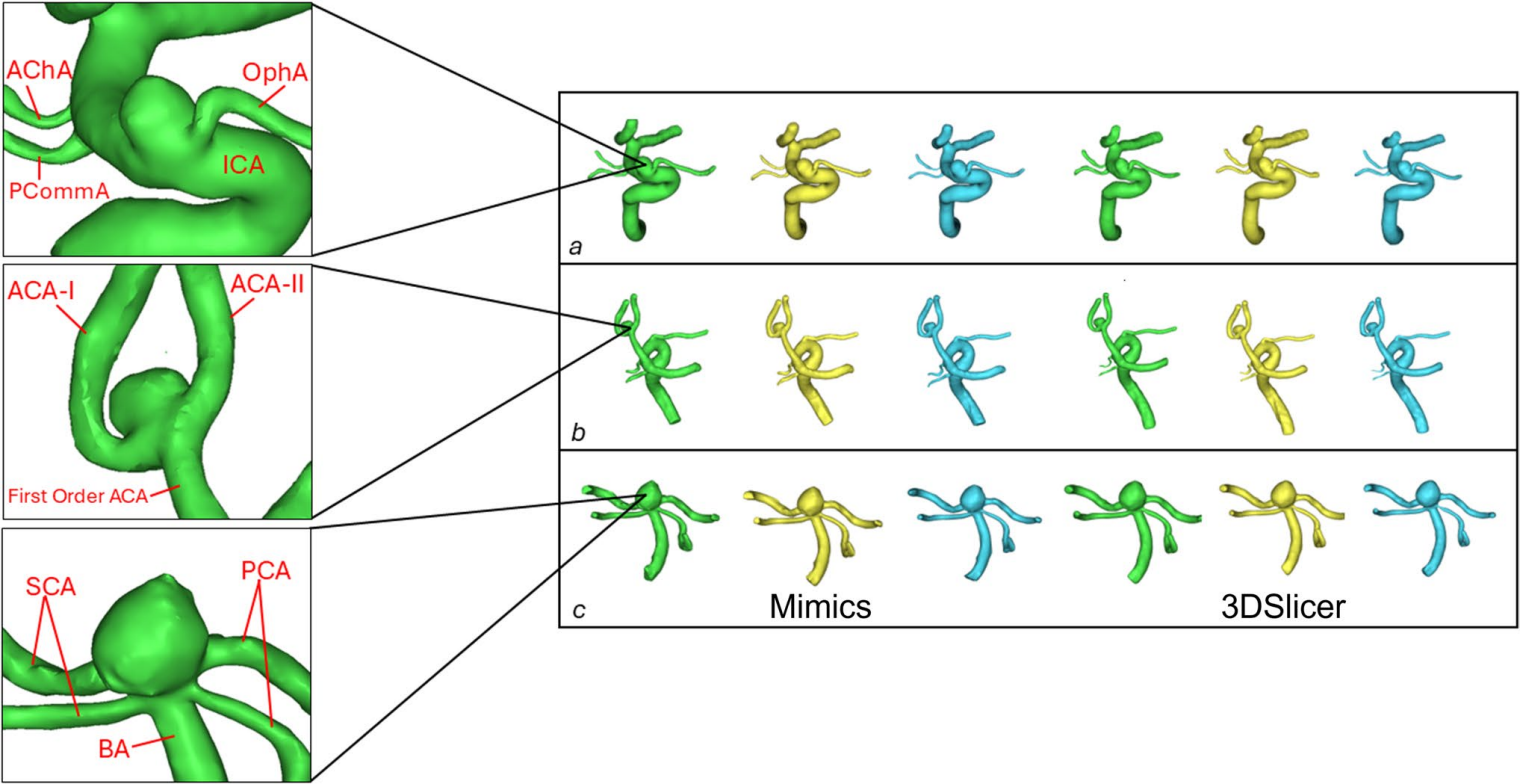
Three complete patient cases each containing six reconstructed IA models; three models from each software in ascending order of lower threshold (i.e., 1000, 1500, 2500 GV):** a**. ICA sidewall aneurysm;** b**. ACA bifurcation aneurysm;** c**. BA bifurcation aneurysm

The majority of aneurysms observed across the 100-patient dataset were categorized into three anatomical subtypes: internal carotid artery (ICA) sidewall aneurysms, anterior cerebral artery (ACA) bifurcation aneurysms, and basilar artery (BA) bifurcation aneurysms, as demonstrated by three typical aneurysms in Fig. 2. For ICA and ACA cases, the ICA, ophthalmic artery (OphA), posterior communicating artery (PCommA), anterior choroidal artery (AChA), middle cerebral artery (MCA), and ACA were identified and preserved. For BA aneurysms, the BA, superior cerebral artery (SCA), and posterior cerebral artery (PCA) were isolated prior to further processing.

After vascular segmentation, noise and minor artifacts were manually removed under the supervision of a postdoctoral researcher with over four years of experience in neurovascular modeling. The cleaned segmentations were then subjected to software-specific smoothing algorithms. In Mimics, Laplacian smoothing was applied with a smoothing factor of 0.5 and 3 iterations. In 3D Slicer, the “Median” smoothing method was used with a kernel size of 1.00 mm. The resulting STL files from both software were imported into 3Matic (Materialise NV, Leuven, Belgium) for post-processing. [9, 10]

### Model Post-processing

The raw vascular models generated in Mimics and 3D Slicer were exported as STL files and imported into 3-Matic for post-processing. To ensure consistency across platforms identical post-processing steps were applied to all models.

First, Laplacian smoothing was performed using a smoothing factor of 0.4 over 10 iterations to generate a primary arterial surface. This was followed by local curvature smoothing (smoothing factor: 0.4) with a characteristic diameter of 0.03 mm to remove small surface irregularities and localized artifacts. The selected diameter was chosen to approximate the minimum mesh resolution typically used in subsequent hemodynamic simulations. Finally, distal vessel segments were trimmed to define consistent inlet and outlet boundaries. For ICA models, trimming was performed near the lacerum segment to preserve a sufficient inlet length for fully developed flow conditions. For all other branches, trimming locations were set at approximately five vessel diameters upstream of the nearest bifurcation. In all cases, trimming planes were defined as perpendicular to the local vessel centerline direction.

To evaluate whether the post-processing workflow influenced aneurysm morphology, a sensitivity analysis was performed on a subset of 18 models. Morphometric measurements were obtained directly from the exported segmentation masks prior to post-processing and compared with measurements obtained after the standardized post-processing pipeline in Materialise 3-matic. Additional details and figures from this analysis are provided in the Supplementary Material.

### Model Measurements and Statistics

**Fig 3:**
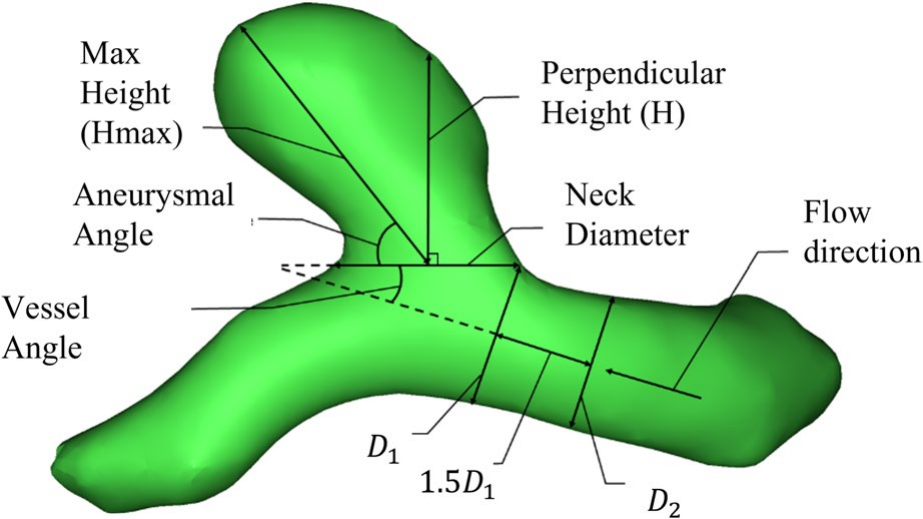
IA morphological parameters

Linear measurements of each model were manually taken using virtual calipers via 3Matic. We juxtaposed the 3D models and manually rotated them to get the same orientation when needed for comparison. Twelve measurements were taken at every artery as well as before and after each bifurcation; after the first six measurements were taken, the model was rotated 90°, relative to the first measurement angle of the given artery or section thereof, and the final six measurements were taken. Each set of twelve measurements was averaged to calculate an average vessel diameter. The morphological parameters of the IA that were measured as demonstrated in Fig. 3, including commonly measured quantities for aneurysm classification such as the neck diameter, perpendicular height (H), and maximum height (H_max_). Quantities used to assess rupture risk as defined by Raghavan et al [11]. and Dhar et al. [12] were also measured as aneurysmal size, vessel angle, aneurysmal angle, as well as D_1a/b_, D_2a/b_, and D_3a/b_ which correspond to the vessel diameter at the neck or branching point (D_1a_, D_2a_, D_3a_) and the vessel diameter 1.5 Da away from D_1a_, D_2a_, and D_3a_ (D_1b_, D_2b_, D_3b_). Aneurysm measurements were taken from a subjectively selected “best” perspective angle for each model; this angle was left to the discretion of each researcher.

Mimics as well as the measurements taken by R1 from the models reconstructed using the 2500 GV threshold were considered baselines for relative difference calculations. Mimics was chosen because it is a proprietary software package whereas 3D Slicer is open source, R1 was chosen due to having the most experience with the software, and 2500 GV was an experienced-based arbitrary choice.

When all measurements were completed, the data was compiled and analyzed against a significance level of α=0.05. Normality was evaluated using the Shapiro-Wilk test, and statistical differences were assessed using paired t-tests and relative percent differences.

Inter- and intra-user variability were assessed using intraclass correlation coefficients (ICC) with corresponding 95% confidence intervals. Inter-user variability was evaluated across all users using the full dataset of aneurysm models while intra-user variability was assessed by having R1 re-measure a representative subset of three varied aneurysm models in three independent sessions conducted on separate days. For intra-user analysis, mean absolute differences and mean percent differences were also calculated.

## Results

To accurately assess the extent to which individual sources of variability affect intracranial aneurysm model reconstruction and evaluation, measurements were analyzed according to the three main factors outlined previously: software platform (Mimics vs 3D Slicer), segmentation threshold (1000, 1500, 2500 GV), and inter-user variability (researchers R1, R2, and R3). When analyzing one of these factors, the other two were held as constants in order to eliminate the potential for compounding error. Mimics, R1, and 2500 GV were also selected as constants for statistical analysis. Meaning, when looking at variability due to software, R1’s measurements of only the 2500 GV threshold were compared between the variables Mimics and 3D Slicer. Similarly, when looking at variability due to threshold, only R1’s measurements from Mimics were compared between the variables 1000, 1500, and 2500 GV. The same principle was applied to looking at evaluation variability due to users i.e., R1, R2, and R3’s measurements of the 2500 GV models made in Mimics.

### Test for Normal Distribution

The Shapiro-Wilk test was used to analyze the data for normal distribution. The results in Table 1 show that no anatomical region achieved a p-value greater than 0.05, therefore, rejecting the null hypothesis of the Shapiro-Wilk test. However, when looking at the histograms and Q-Q plots of the data (see Supplemental Files), the data appears normal, and further skew and kurtosis were also calculated. The skew and kurtosis were within reasonable ranges for all anatomical regions (− 1 to 1 and − 2 to 2, respectively) and showed that all anatomical regions can be generally considered normally distributed.

**Table 1:** Results of the Shapiro-Wilk test for normal distribution as well as data skew and kurtosis for context

	Mimics				3D slicer			
	Shapiro W	P-value	Skew	Kurtosis	Shapiro W	P-value	Skew	Kurtosis
Inlet	0.99	0.030	− 2.1	− 0.23	0.99	0.014	− 0.22	− 0.12
Outlets	0.97	< 0.001	0.7	0.23	0.96	< 0.001	0.72	0.35
Neck	0.98	< 0.001	0.5	− 0.12	0.97	< 0.001	0.48	− 0.18
H	0.95	< 0.001	0.9	1.1	0.95	< 0.001	0.95	1.2
Hmax	0.94	< 0.001	0.9	0.80	0.94	< 0.001	0.96	0.90
Size	0.94	< 0.001	1.0	1.4	0.94	< 0.001	0.96	1.2

### IA Reconstruction Variability

Models generated in 3D Slicer were consistently smaller than those generated in Mimics across all six anatomical regions as seen in Table 2. The average percent differences ranged from − 1.8 to − 4.3%. The largest difference was observed within outlet diameters, where Mimics models averaged 2.4 ± 0.2 mm compared to 2.3 ± 0.8 mm in 3D Slicer (− 4.4%, p < 0.001 based on the paired t-test). Similar trends were observed in neck measurements which showed differences of − 4.2% (p < 0.001). Among all other regions, the reduction, while smaller, remained statistically significant with a  − 1.8, − 1.6, − 1.3, and − 1.5% change for inlet diameter, H, Hmax, and aneurysmal size, respectively and all p < 0.05.

**Table 2:** Statistical analysis of geometric difference due to software

	Mimics	3D slicer	3D Slicer v Mimics	
	2500 GV Average D (mm) ± sd	2500 GV Average D (mm) ± sd	%Diff_2500 GV (%)	P-value
Arteries				
Inlet	4.7 ± 0.7	4.6 ± 0.7	− 1.8	< 0.001
Outlets	2.4 ± 0.2	2.3 ± 0.8	− 4.4	< 0.001
Aneurysm				
Neck	4.9 ± 1.4	4.7 ± 1.4	− 4.2	< 0.001
H	4.9 ± 2.2	4.8 ± 2.2	− 1.6	< 0.001
Hmax	5.4 ± 2.5	5.4 ± 2.5	− 1.3	< 0.001
Size	5.6 ± 2.4	5.5 ± 2.4	− 1.5	< 0.001

These differences show consistently smaller dimensions created by 3D Slicer relative to Mimics likely stemming from differences in their edge detection algorithms and voxel intensity thresholding strategies. All six anatomical regions demonstrated statistically significant differences, p < 0.05, strongly suggesting that the software platform itself can introduce systematic geometric variability in IA model reconstruction.

Decreasing the threshold resulted in larger measurements across all anatomical regions as seen in Table 3. The most notable changes again occurred within outlet diameter measurements where decreasing the threshold from 2500 GV (2.4 ± 0.8 mm) to 1000 GV (2.8 ± 0.8 mm) produced a relative increase of 15.9% (p < 0.001). All other regions exhibited a similar increase as threshold decreased from 2500 to 1000 GV. Inlets were measured, on average, 8.1% larger (p < 0.001); neck measurements were 11.3% larger (p < 0.001); H was found to be 9.1% larger (p < 0.001); Hmax measured 9.4% larger (p < 0.001); and aneurysmal size was 9.2% larger on average (p < 0.001).

**Table 3:** Statistical analysis of variability between thresholds

	1000 GV	1500 GV	2500 GV	1000v2500 GV		1500v2500 GV	
	Average D (mm) ± sd	Average D (mm) ± sd	Average D (mm) ± sd	%Diff (%)	P-value	%Diff (%)	P-value
Arteries							
Inlet	5.0 ± 0.8	4.9 ± 0.7	4.7 ± 0.7	8.1	< 0.001	4.6	< 0.001
Outlets	2.8 ± 0.8	2.6 ± 0.8	2.4 ± 0.8	15.9	< 0.001	9.6	< 0.001
Aneurysm							
Neck	5.4 ± 1.4	5.2 ± 1.4	4.9 ± 1.4	11.3	< 0.001	5.6	< 0.001
H	5.2 ± 2.2	5.0 ± 2.2	4.9 ± 2.2	9.1	< 0.001	3.8	< 0.001
Hmax	5.9 ± 2.5	5.7 ± 2.5	5.4 ± 2.5	9.4	< 0.001	4.6	< 0.001
Size	6.1 ± 2.4	5.8 ± 2.4	5.6 ± 2.4	9.2	< 0.001	3.7	< 0.001

**Fig 4:**
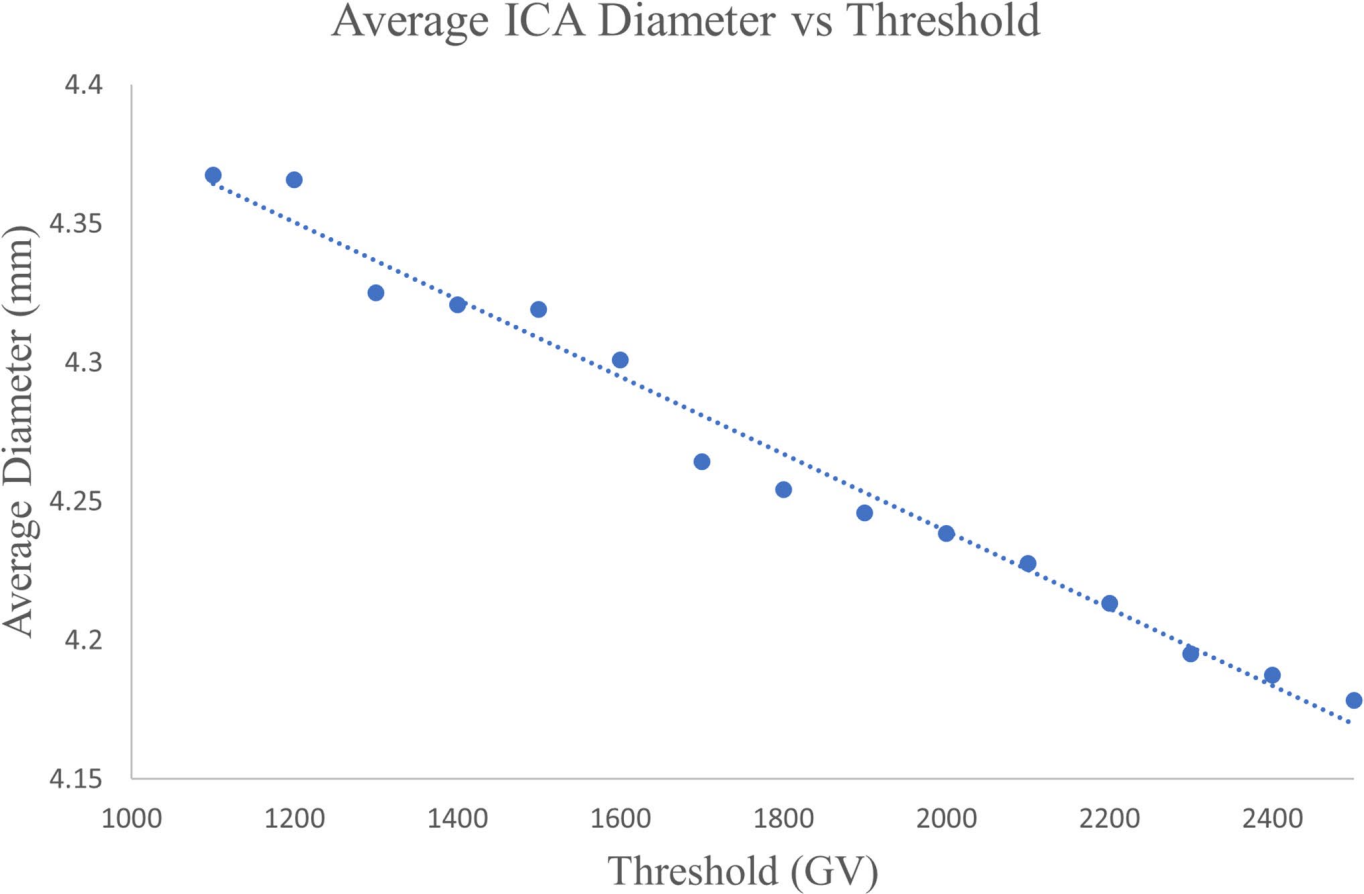
Average diameter of ICA for a patient varies with the lower threshold values for model reconstruction

Even moderate decreases in threshold, such as from 2500 to 1500 GV, resulted in, lesser, but still significant increases in nearly every region. For example, Hmax showed a 4.6% change (p < 0.05) and the diameter of outlets increased by 9.6% (p < 0.05). The same trend was seen across all other regions and all p-values < 0.05. These results strongly suggest that threshold selection is a critical determinant of model geometry with lower thresholds consistently yielding more expansive segmentations due to inclusion of lower-intensity voxels. To further investigate the relationship between thresholds and the model dimensions, 15 models of the ICA of a single patient were created in Mimics to find the fit for the trend of data as threshold changes. The first model was made with a lower threshold of 1100 GV, and a step size of 100 GV was adopted to increase the threshold until a final model with the threshold of 2500 GV. Each model was processed as described in *Model Reconstruction*, and measurements were taken in the same manner as those done for the 100 patient data set. It was found that a linear model fits the decreasing diameter of the ICA as lower threshold increases, as demonstrated in Fig. 4. The linear curve fit equation is determined as Eqn (1) with $R^{2} = 0.98$:

1 D=4.51-0.0001×GV

where D is the averaged diameter of fifteen ICA models, and GV is the grey value of lower threshold for model reconstruction in Mimics.

Sensitivity analysis demonstrated minimal bias between measurements obtained before and after post-processing. Bland–Altman analysis showed small differences with narrow limits of agreement indicating that the standardized post-processing workflow introduced negligible geometric variation relative to the segmentation-related differences examined in this study (See Supplemental Files).

### IA Model Evaluation Variability

As shown in Table 4, measurements from R2 closely matched those of R1 across nearly all geometric variables. Inlet and outlet diameters differed by only 2.6% and 0.7%, respectively, and differences in aneurysmal measurements only ranged from − 1.0 to 0.1%. These small differences translated to statistically insignificant p-values.

**Table 4:** Statistical analysis of variability between users including intraclass correlation coefficients (ICC) with corresponding 95% confidence intervals

	R1	R2	R3	R2vR1		R3vR1			
	Average D 2500 GV (mm) ± sd	Average D 2500 GV (mm) ± sd	Average D 2500 GV (mm) ± sd	% Diff (%)	P-value	% Diff (%)	P-value	ICC	95% CI
Arteries									
Inlet	4.7 ± 0.7	4.8 ± 0.7	4.8 ± 0.8	2.6	< 0.001	3.7	< 0.001	0.966	0.947–0.977
Outlets	2.4 ± 0.8	2.4 ± 0.8	2.4 ± 0.8	0.7	0.30	0.90	0.033	0.986	0.982–0.989
Aneurysm									
Neck	4.9 ± 1.4	4.9 ± 1.4	5.0 ± 1.5	− 0.3	0.60	2.1	0.052	0.981	0.974–0.987
H	4.9 ± 2.2	4.8 ± 2.1	4.7 ± 2.2	− 1.0	0.49	− 3.4	0.066	0.978	0.969–0.985
Hmax	5.4 ± 2.5	5.5 ± 2.4	5.4 ± 2.4	0.1	0.91	0.30	0.81	0.987	0.981–0.991
Size	5.6 ± 2.4	5.6 ± 2.4	6.9 ± 2.8	− 0.8	0.40	22.7	< 0.001	0.940	0.796–0.974

In contrast, R3's results began to diverge more meaningfully. Inlet diameter was inflated by 3.7% compared to R1, and outlet diameter showed a 0.9% increase. While aneurysmal quantities such as Hmax remained stable (0.3% difference), others showed substantial variability. The most dramatic was aneurysmal size which averaged 6.9 mm for R3 versus 5.6 mm for R1; a 22.7% increase that was highly significant (p < 0.001).

These results strongly suggest that, with proper training time and experience, differences due to users becomes statistically insignificant. R3 had one month of experience with the software and three out of six anatomical regions showed highly statistically significant differences while R2 had six months of experience and only one anatomical region showed statistically significant differences when compared to R1 (Table 4).

### Inter/Intra-rater Variability

Inter-rater reliability demonstrated excellent agreement across all parameters (ICC range 0.940–0.987) with consistently high 95% confidence intervals (Table 4). Intra-rater repeatability demonstrated minimal variability across all metrics with an overall mean absolute difference of 0.012 mm and mean percent difference of 0.44%. Intra-rater ICC values approached unity for all evaluated parameters indicating excellent repeatability of manual measurements (Table 5).

**Table 5:** Intra-rater variability analysis showing mean absolute differences, percent differences, and ICC with 95% confidence intervals for each metric

	Mean absolute difference (mm)	Mean percent difference (%)	ICC	95% CI
Arteries				
Inlet	0.009	0.22	0.999	0.999–1.000
Outlets	0.010	0.50	0.999	0.999–1.000
Aneurysm				
Neck	0.011	0.27	0.999	0.999–1.000
H	0.011	0.28	0.999	0.998–0.999
Hmax	0.009	0.22	0.999	0.999–1.000
Size	0.004	0.09	0.999	0.999–1.000
Overall	0.012	0.44		

### Segmentation Time

Based on the experience of R1, segmentation using Mimics generally required approximately 20-30 minutes per case, whereas segmentation in 3D Slicer required approximately 15–25 minutes per case. These estimates reflect typical workflow durations under consistent conditions but may vary depending on user experience and case complexity.

### Vessel and Aneurysm Angle

It is worth noting that the data for vessel and aneurysm angle measurements does not follow a normal distribution, and, therefore, cannot be statistically analyzed for relevance. The angle at which Hmax is found as well as the angle at which the neck of the aneurysm coincides with the midline of the parent artery is completely random. More so than other aneurysm related quantities, vessel and aneurysm angle are extremely dependent on viewing angle. As previously mentioned, the viewing angle was left up to the discretion of the individual researchers as defining an objective “perfect” viewing angle would be very difficult and not within the scope of this study. Furthermore, being angular quantities rather than distance, there is no reason for them to change consistently from one threshold to another or from one software to another.

## Discussion

Accurate reconstruction and evaluation of intracranial aneurysm models is critical for numerous clinical and research applications including surgical planning, device development, and hemodynamic simulations. The fidelity of these reconstructions depends heavily on the segmentation process which is influenced by a variety of factors including threshold settings, the segmentation software used, and the interaction of the operator with the tools. While previous studies have primarily focused on validating the accuracy of IA models generated using individual segmentation tools, comparatively fewer have sought to dissect the variability introduced by different segmentation workflows and user interactions. For instance, Firouzian et al. used geodesic active contours to segment aneurysms in computational tomography angiography (CTA) demonstrating good agreement with manual segmentations but were limited by a small sample size (15 aneurysms) and reliance on a single segmentation technique [1]. Acar et al. validated time-of-flight magnetic resonance angiography (MRA)–derived 3D models by comparing them to rescanned 3D prints reporting deviations of ~ 0.3 mm; however, their study was limited to MRA data and large aneurysms which reduces applicability [2]. Several researches focused on the comparison between manual thresholding or gradient edge detection method with ground truth measurements using phantom models [3, 4]. Mantilla et al. compared STL meshes to their physical 3D-printed counterparts and found < 0.21 mm surface deviations for 80% of meshes’ nodes [5], but the study only included five patients and did not consider cross-software differences. Sen et al. analyzed the impact of segmentation algorithm choice (region-growing vs. Chan–Vese level-set) on aneurysm volume, reporting up to 24% volume variation between methods [6]; then, they further compared the wall shear stress (WSS) in the hemodynamic analysis with the averaging difference of 126.4% for the lowest volumes of WSS [7]. However, this study was limited by the absence of physical validation or cross-modality comparison. Mandolini et al. compared Mimics and 3D Slicer for femoral head segmentation and found consistent but modest differences (<3%) in extracted diameters [8], though the study did not examine cerebral vasculature or quantify operator variability. Tatit et al. compared volume measurements across manual and semi-automated tools (e.g., RapidAI, ITK-SNAP) [13], finding biases of up to 25 mm^3^, but their method mixed multiple imaging modalities (i.e., three-dimensional rotational angiography, MRA), and software settings were not standardized. Similarly, Zhu et al. used a deep learning model (3D U-Net) and reported Dice scores around 0.80 and mean surface deviations of 0.35 mm. [14] Although they highlighted general segmentation uncertainty, the work did not focus on commonly used commercial tools like Mimics or 3D Slicer and lacked comparison with manual baselines.

In this study, the influence of segmentation threshold, software platform, and operator variability on the geometry and subsequent evaluation of IA models across arteries as well as relevant aneurysm measurements was systematically examined. A total of 600 models from 100 patients were analyzed, and statistical comparisons were made within and across these variables. Consistent trends emerged across nearly all regions allowing for several key observations.

First, the comparison between software tools revealed systematic geometric differences. Across all regions, models generated in 3D Slicer were consistently smaller than those produced in Mimics with average differences ranging from − 1.3 to − 4.4% and statistically significant in all regions. This size discrepancy may be influenced, in part, by differences in how each software handles DICOM intensity metadata particularly the rescale intercept. In the source DICOM data, a rescale intercept of approximately − 1024 is specified by tag (0028,1052). When probing identical voxel locations across platforms, a consistent offset of approximately 1024 intensity units was observed with values appearing higher in Mimics compared to 3D Slicer. This indicates that the intensity values used for segmentation are represented differently between software environments despite originating from the same underlying imaging data. With this difference in mind, a comparison between the 1500 GV threshold in 3D Slicer and the 2500 GV threshold in Mimics was done as presented in Table 6. The results generally showed smaller differences across anatomical regions than comparing the same threshold between software, but p-values continued to show the differences to be statistically significant in most regions. Other factors that could influence the geometric differences include potential differences in edge detection algorithm. These findings are supported by existing comparative work such as Mandolini et al. [8] who reported similar volume discrepancies between Mimics and 3D Slicer in femoral head segmentation.

**Table 6:** Statistical analysis accounting for differences in DICOM metadata interpretation

	3D slicer	Mimics	3D slicer v mimics	
	Average D 1500 GV (mm) ± sd	Average D 2500 GV (mm) ± sd	%Diff (%)	P-value
Arteries				
Inlet	4.8 ± 0.7	4.7 ± 0.7	1.7	< 0.001
Outlets	2.5 ± 0.8	2.4 ± 0.8	3.9	< 0.001
Aneurysm				
Neck	4.9 ± 1.4	4.9 ± 1.4	− 0.13	0.84
H	5.0 ± 2.2	4.9 ± 2.2	1.9	< 0.001
Hmax	5.5 ± 2.5	5.5 ± 2.5	1.6	< 0.001
Size	5.7 ± 2.4	5.6 ± 2.4	2.1	< 0.001

Second, the effect of segmentation thresholds on model geometry was apparent in both software platforms. As the threshold decreased from 2500 to 1000 GV, average diameters consistently increased. This trend is consistent with prior literature including the findings of Firouzian et al. [1] and Acar et al. [2] which documented threshold-dependent reductions in model dimensions and highlighted the impact of intensity-based segmentation boundaries. In the observed dataset, the percentage increase between 2500 and 1000 GV ranged from approximately 3.8 to 15.9% depending on the anatomical region (p < 0.05 in all regions).

Third, inter-operator variability can be a significant contributor to model evaluation uncertainty. The statistical trends strongly suggest that, as experience with software packages increases, statistically significant differences between users decreases. Half of the anatomical regions measured by R3 showed statistically significant differences (Inlet, Outlets, and Size) when compared to the same regions measured by R1 while only one of the anatomical regions measured by R2 (Inlet) showed statistically significant differences. This could suggest individual interpretation of vessel boundaries, especially in regions with less distinct gradients, can substantially affect model outcomes, but, with time and experience, these interpretations normalize. While such variability has been noted in other studies [6, 13], our results provide a direct quantification of its impact.

Finally, this work reinforces the importance of standardizing segmentation workflows in IA modeling. While many of the observed differences were statistically significant, their direct clinical significance in terms of diagnosis or treatment planning may be limited particularly for differences in the order of 0.1 mm. However, importantly, small geometric differences have large relevance in downstream applications such as CFD where hemodynamic parameters, including wall shear stress and flow patterns, can be very sensitive to subtle changes in vascular geometry. Therefore, even minor variations introduced during segmentation and reconstruction may influence subsequent analyses as demonstrated by Sen et al. [7] As segmentation tools continue to evolve further work is needed to benchmark their performance against traditional methods and to reduce variability in model generation. Recent advances in artificial intelligence (AI), particularly deep learning–based approaches, have shown positive performance in vascular segmentation tasks. Architectures such as U-Net have become widely adopted for biomedical image segmentation [15] and subsequent studies have demonstrated their effectiveness in cerebrovascular applications [16]. Furthermore, emerging work has extended these approaches beyond segmentation to enable full reconstruction of patient-specific three-dimensional vascular models from imaging data [17]. However, AI-based methods introduce new sources of variability including dependence on training data, model architecture, and generalizability across imaging modalities. As such, while AI has the potential to reduce operator dependency and improve efficiency, it does not fully eliminate variability in model generation.

The current results demonstrate that the geometry of intracranial aneurysm models is significantly affected by the choice of segmentation threshold and software platform, and IA model evaluation can be affected by operator. These factors contribute meaningful uncertainty to model dimensions and should be carefully considered in both clinical and research applications. While validation of individual tools remains essential, our findings highlight the importance of examining the entire segmentation pipeline when evaluating IA model fidelity.

**Fig 5:**
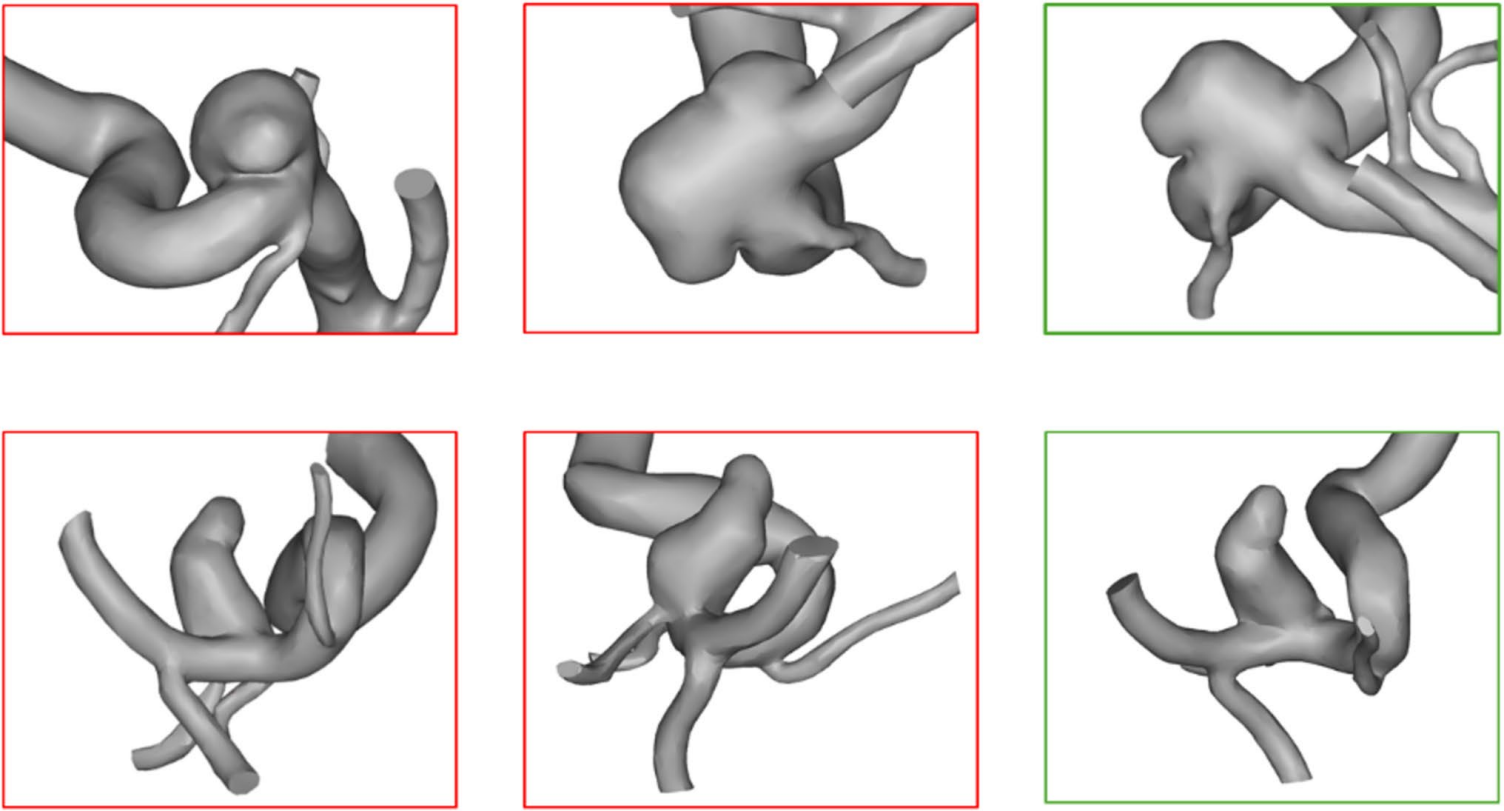
Two examples of cases that caused discrepancies between users due to aneurysmal shape or position and their proper orientation for measurement

It is worth mentioning that certain cases had abnormally shaped or awkwardly positioned aneurysms as illustrated in Fig. 5. Some of these positionings and abnormalities caused significant differences in measurements taken by the three researchers due to the fact that, as previously mentioned, the viewing angle for aneurysmal measurements was left up to the discretion of each individual. In the pursuit of adding as much to the standardization of IA model reconstruction and evaluation as possible, the following guidelines are proposed for measuring extremely abnormally shaped or strangely positioned aneurysms: 1. The neck of the aneurysm should be oriented parallel to the bottom of the software UI screen, 2. The vertex of the aneurysm should be oriented upward when considering the neck of the aneurysm as a baseline for direction, 3. The aneurysm should be angled as to maximize Hmax, and 4. The aneurysm should be angled as to maximize the amount of the parent artery that is visible in order to maximize the diameter of the neck of the aneurysmal sac.

This study systematically evaluated how segmentation threshold, software platform, and user interaction influence the geometry of intracranial aneurysm (IA) models across eight anatomical quantities. The findings confirm that each of these factors introduces measurable variability into model reconstruction. Notably, 3D Slicer produced slightly smaller models than Materialise Mimics across all regions when comparing the same thresholds (up to a difference of − 4.8%). However, accounting for the difference in DICOM metadata interpretation, 3D Slicer created slightly larger models across most regions. Decreasing segmentation thresholds consistently increased measured diameters (up to 15.9%), and inter-operator variability can certainly be a source of uncertainty in evaluation, however, with sufficient exposure to the software, this variability tends to normalize. These statistically significant discrepancies underscore the importance of standardizing segmentation protocols particularly in computational research and clinical workflows involving IA models. Given the sensitivity of downstream analyses, such as hemodynamic simulation to geometric input, careful consideration of segmentation parameters is essential to ensure consistency and reproducibility. This work supports the hypothesis that segmentation-related variables contribute substantially to geometric uncertainty in IA modeling and highlights the need for transparent, validated pipelines in both research and clinical practice.

There are several notable limitations to this study. First, although the dataset includes 600 models, it is constrained by the use of only three segmentation thresholds and two software platforms. Additional thresholds or inclusion of machine learning–based segmentation methods might yield further insight. Second, while the three researchers followed a standardized protocol, subtle biases in interpretation and manual refinement likely introduced variability. This is inherent to most segmentation tasks but should be acknowledged when interpreting inter-operator differences. Third, the study did not incorporate a ground truth model—either from histology, physical phantoms, or expert consensus. As such, the analysis quantifies relative differences rather than absolute errors. In vitro experiments with 3D printed models will be implemented in the near future to study the accuracy of model reconstructions using a ground truth model. Meanwhile, the next stage of the study will focus on hemodynamic discrepancies caused by the morphology variability due to software and thresholds

## Supplementary Information

Below is the link to the electronic supplementary material.

Supplementary file1 (DOCX 1339 KB)

## Data Availability

The data involved in this research is available upon request addressed to the corresponding author.
