# Supplementary material for: Sources of Variability in Intracranial Aneurysm Model Reconstruction and Evaluation: A Systematic Investigation
Source: Cardiovasc Eng Technol. 2026 May 7;17(4):414–24. doi: 10.1007/s13239-026-00841-1 (PMC13442564; doi:10.1007/s13239-026-00841-1)
Supplement: Supplementary file 1 [file 13239_2026_841_MOESM1_ESM.docx]

**Supplemental Files**

**Sources of Variability in Intracranial Aneurysm Model Reconstruction and Evaluation: A Systematic Investigation**

**Methods**

All patient DICOM datasets were acquired from digital subtraction angiography (DSA) scans performed at Miami Valley Hospital (Premier Health) in Dayton, OH, USA. Each dataset was anonymized and imported into both Materialise Mimics (v25.0) and 3D Slicer (v5.6.2) without pre-processing. DICOM metadata from both software platforms was extracted using the “Export DICOM Tags” function in Mimics and the “Copy All Metadata” function in Slicer.

All segmentations were performed using global thresholding with lower bounds of 1000, 1500, or 2500 gray values (GV). No additional image filters (e.g., smoothing or denoising) were applied before segmentation.

It is worth noting that imperfections when performing DSA scanning created surface imperfections in some cases. While there is a possibility that some of these imperfections were accurate to the anatomy of the given patient, it was left up to the discretion of R1 and R2 to assess vessel geometry and make a judgment between reasonable anatomical discrepancies versus surface imperfections caused by data collection. A large factor influencing this decision was the potential of the imperfection to have a negative effect on future hemodynamic CFD analysis.

**Histograms and Q-Q Plots for Normal Distribution**

Fig. S1-S6 show the histograms and Q-Q plots mentioned in *Test for Normal Distribution* used to analyze and confirm normal distribution after the Shapiro-Wilk test was performed. All graphs and plots visually suggest normally distributed data.

**Figure S1.** Histograms and Q-Q Plots of Inlet Diameter Data

**Figure S2.** Histograms and Q-Q Plots of Outlet Diameter Data.

**Figure S3.** Histograms and Q-Q Plots of Neck Diameter Data.

**Figure S4.** Histograms and Q-Q Plots of Aneurysmal Height Data.

**Figure S5.** Histograms and Q-Q Plots of Maximum Aneurysmal Height Data.

**Figure S6.** Histograms and Q-Q Plots of Aneurysmal Size Data.

**Comparison of Models**

Model reconstruction was split between R1 and R2. R1 reconstructed 79 of the 100 patients while R2 reconstructed 21. Both individuals were well trained on this matter and 20 raw models from 10 cases were reconstructed by both R1 and R2 overlayed on each other to show differences. Models from six patients were shown in Fig. S7. These models are from the 2500 GV threshold of both Mimics and 3DSlicer. R1’s model is highlighted in green. Main differences occurred in overall vessel length rather than geometry. This is a subjective choice that is standardized across all models in a given case before the local curvature smoothing in 3Matic.

**Figure S7.** Comparison of 2500 GV Models for six patients reconstructed by R1 (Green) and R2 (Gray) in Mimics (Left) and 3DSlicer (Right).

**Sensitivity Analysis**

To evaluate whether post-processing influenced aneurysm morphology, a sensitivity analysis was performed on a representative subset of 18 models. For these models, morphometric measurements were obtained directly from the exported segmentation masks prior to post-processing and compared with measurements obtained after the standardized post-processing workflow in Materialise 3-matic. Bland–Altman analyses and corresponding scatter plots were generated for both linear and angular parameters. Bland–Altman analysis showed small differences with narrow limits of agreement indicating that the standardized post-processing workflow introduced negligible geometric variation relative to the segmentation-related differences examined in this study.

**Figure S8:** Bland–Altman analysis and scatter plot comparing linear measurements obtained before and after post-processing.

**Figure S9:** Bland–Altman analysis and scatter plot comparing angular measurements obtained before and after post-processing.
